# Bayesian network-driven clustering analysis with feature selection for high-dimensional multi-modal molecular data

**DOI:** 10.1038/s41598-021-84514-0

**Published:** 2021-03-04

**Authors:** Yize Zhao, Changgee Chang, Margaret Hannum, Jasme Lee, Ronglai Shen

**Affiliations:** 1 Department of Biostatistics, Yale University, New Haven, CT USA; 2 Department of Biostatistics, Epidemiology and Informatics, University of Pennsylvania Perelman School of Medicine, Philadelphia, PA USA; 3 Department of Epidemiology and Biostatistics, Memorial Sloan-Kettering Cancer Center, New York, NY USA

**Keywords:** Cancer, Computational biology and bioinformatics

## Abstract

Multi-modal molecular profiling data in bulk tumors or single cells are accumulating at a fast pace. There is a great need for developing statistical and computational methods to reveal molecular structures in complex data types toward biological discoveries. Here, we introduce *Nebula*, a novel Bayesian integrative clustering analysis for high dimensional multi-modal molecular data to identify directly interpretable clusters and associated biomarkers in a unified and biologically plausible framework. To facilitate computational efficiency, a variational Bayes approach is developed to approximate the joint posterior distribution to achieve model inference in high-dimensional settings. We describe a pan-cancer data analysis of genomic, epigenomic, and transcriptomic alterations in close to 9000 tumor samples across canonical oncogenic signaling pathways, immune and stemness phenotype, with comparisons to state-of-the-art clustering methods. We demonstrate that Nebula has the unique advantage of revealing patterns on the basis of shared pathway alterations, offering biological and clinical insights beyond tumor type and histology in the pan-cancer analysis setting. We also illustrate the utility of Nebula in single cell data for immune cell decomposition in peripheral blood samples.

## Introduction

Recent technological advances have allowed the collection of high-dimensional, multiscale molecular data using genome-wide platforms that assayed DNA (exome sequencing, DNA methylation, and copy number profiling) and RNA (mRNA and microRNA sequencing) expression in large tumor cohorts^1^ as well as in single cell populations^2,3^. There is a great need for advanced statistical and computational methods and algorithms to facilitate scientific discoveries from mining these accumulating data sources. In this study, we focus on cross-modality integration and clustering analysis with two major applications: tumor subtype stratification and deconvolution of single cell subpopulation.

In tumor profiling studies, integrative clustering analysis allows patient stratification into subtypes that bring great potential to personalize cancer diagnosis and treatment^4,5^. The recent pan-cancer integrative molecular classification study has revealed groupings of tumor samples primarily organized by histology, tissue type, or anatomic origin^1^. Nevertheless, recent oncology drug development landscape is dominated by efforts on targeting genomic alterations independent of tumor histology^6,7^. Examples include PARP inhibitors targeting HR-deficiency and immunotherapies approved for pan-cancer indication for MSI subtype. Therefore, a statistical and computational tool that allows discovery of subgroups of patient population on the basis of shared pathway alterations will enrich novel designs of modern clinical trials. In single cell studies, clustering analysis has been developed for spatial reconstruction, cell identity classification, and clonal decomposition^8–11^. With recent advances in multi-modality profiling of single cells^2,3,12,13^, integrative clustering algorithms with the incorporation of network priors will allow more in-depth understanding of cell heterogeneities and interactions.

Clustering models that incorporate multiple data types have been recently developed under joint latent variable approaches^14,15^, kernel- or graph-based integration^16,17^ and Bayesian parametric or nonparametric mixture models^9,18^. Although most of them are capable to characterize a certain degree of concordance and heterogeneity across data types, there are still several main challenges. First, it is computationally imperative as well as biologically important to simultaneously identify subgroups and pinpoint the core sets of biomarkers that characterize the subgroups with probabilistic model inference. Secondly, there is a need to incorporate knowledge on biological functions (e.g. molecular pathways and regulatory networks) within and across molecular modalities for a more interpretable and useful classification of tumor and cell populations. It is particularly important to delineate molecular heterogeneity and biological understanding beyond cell of origin. Finally, scalable implementation is crucial for clustering analysis of modern multi-model data sets across tens of thousands of patient samples or single cells. To address these challenges, we aim to develop a high-dimensional clustering method that incorporates biological network information across different data modalities. We present Nebula (Network-based multi-modal clustering analysis), a novel Bayesian network-based clustering analysis for multi-modal integration and clustering with feature selection, and compare its performance in pan-cancer tumor profiling and single cell transcriptome sequencing data to state-of-the-art clustering algorithms including iCluster^14^ and Suerat^8^.

## Results

### Nebula: method overview

**Figure 1:**
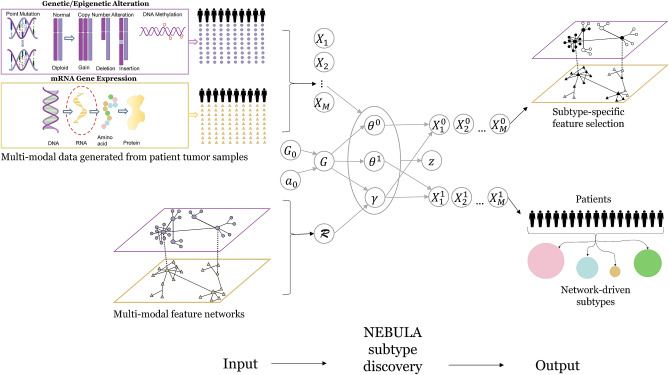
Nebula, a multimodal integrative clustering framework using a Bayesian nonparametric Dirichlet process mixture (DPM) model for simultaneous high-dimensional clustering and feature selection, with biological networks within and between data modalities incorporated as prior knowledge via graph models.

The main analytical objective is to jointly cluster patients based on molecular similarity within and across modalities, and identify associated biomarkers that capture the key characteristics for each subgroup. Under a Bayesian nonparametric Dirichlet process mixture (DPM) model, Nebula is capable to interactively learn group label and molecular signature through the guidance of biological network information. Specifically, as shown in Fig. 1, given the collected *M*-modality data $X_1,\dots ,X_M$ of diverse data types (e.g. Normal, Bernoulli), we first introduce a biomarker- and individual-specific indicator variable $\gamma \in \{0,1\}$ to determine whether the biomarker makes significant contribution to cluster the individual into a particular subtype. Given the value of $\gamma$, each observation is partitioned into a cluster-active set and a cluster-inactive set, with $\varvec{\theta }^0$ and $\varvec{\theta }^1$ representing the associated parameter sets in the null and alternative distributions. The null distributions are cluster-unrelated, and we assign noninformative Bayesian priors on $\varvec{\theta }^0$ to remove unwarranted constrain. While the alternative distributions provide information to define clusters, and we introduce cluster-specific distribution for $\varvec{\theta }^1$ with additional hyper-priors. To further incorporate biological information, we summarize molecular pathways, biological and regulatory networks within and between modalities into indirect graphs with biomarkers as nodes and their connections as edges. Based on the graph, we smooth over indicator set $\varvec{\gamma }=\{\gamma \}$ using multi-modal Ising model under sparse parameters $\varvec{\eta }$ and smooth parameters $\varvec{\nu }$ to induce a coupling effect among selection status of connected biomarkers. Eventually, the joint distribution of $(\varvec{\gamma }, \varvec{\theta }^1, \varvec{\theta }^0)$ discriminates the subjects by clusters (with cluster label *z*) under a mixture of null and alternative distributions, and it follows a distribution *G*, which samples from a DPM with base measure $G_0$ and concentration parameter $\alpha _0$. The discrete nature of DPM realizes a distribution-based grouping effect, where each group is defined only under its specific active biomarker set across modalities denoted as $(X_1^1,\dots ,X_M^1)$. Under our joint modeling framework, the biomarker associated parameters and selection indicators for all data modalities are simultaneously estimated and clustered under the guidance of existing knowledge within and between data modalities, leading to substantially improvements on biological interpretations and statistical power. It is also worth noting that neither pre-specified cluster number nor high-dimensional biomarker screening is required in our model, which ensures the practical superiority of Nebula over existing best practices.

Intensive computation is always a challenging issue for Bayesian modeling particularly for high-dimensional integrative analysis. To allow feasible implementation of Nebula, besides Markov chain Monte Carlo (MCMC), we also develop a computationally efficient variational Bayes (VB) algorithm to approximate posterior distribution. We have done extensive experiments to test the model performance under both MCMC and VB, and conclude that compared with MCMC, our proposed VB algorithm manages to speed-up posterior computation in almost a hundred-fold in the scale of our numerical experiments with minimal sacrifice in clustering and feature identification performance. Technical details of the model and the VB algorithm implementation can be found in Online Method. An open-source software implementation of Nebula is available on GitHub (https://github.com/nebula-group/nebula).

Scalable implementation is crucial for clustering analysis of modern multi-model data sets across tens of thousands of patient samples or single cells. To illustrate the computation efficiency, we conducted a simulation experiment to assess the computational time for the algorithm. Specifically, we perform simulations under sample size 1000 or 5000. Under each sample size, we consider three different feature dimensions with *p* = 1000; 10,000; 100,000 and two simulated data modalities. We assume 10% of the features to be signals which divide all the subjects into three groups. Under each scenario, we generated the first data type from Normal distributions with means $-2$, 2 and 6 and standard deviation 0.1; and the second data type from Bernoulli distributions with probabilities 0.2, 0.5 and 0.8. For noise part, we simply generated them from a standard Normal distribution. Based on the simulation setting above, Nebula can fully uncover the group label for all the subjects under each scenario. The computational cost in seconds under R implementation, 3.4 GHz CPU, 8 GB Memory, Windows System are shown in Table 1. As Table 1 shows, for a dataset of 5000 samples with *P* = 100,000 features, the algorithm can finish within a few hours, which is highly feasible and competitive in computational speed for a Bayesian algorithm.

**Table 1:** Assessment of running time (shown in seconds) for Nebula using simulated datasets of varying sizes.

	P = 1000 (s)	P = 10,000 (s)	P = 100,000 (s)
N = 1000	4	152	9120
N = 5000	17	441	23,254

N is the number of samples and P is the number of features.

### Nebula analysis of the TCGA pan-cancer dataset

#### Data description and model fitting

Our analyses include 8855 solid tumor samples across 31 solid tumor types in the TCGA PanCancer Atlas collection. DNA alterations (point mutation, copy number alterations, DNA methylation) detected from whole-exome sequencing and 450k DNA methylation array, mRNA gene expression from mRNA-seq platforms, along with clinical annotation are available for each sample. We format the data into two separate gene-by-sample matrices for DNA alteration and mRNA expression respectively. The DNA matrix annotates individual genetic alterations for each gene by integrating across different data types at the DNA level as described in Sanchez-Vega^19^. Specifically, alterations are classified into activating events (hotspot missense mutations, copy number amplifications or fusions) associated with oncogenes and inactivating events (truncating mutations, deletions, promoter DNA methylations) associated with tumor suppressor genes. For the DNA data matrix, we focus on a total of 187 cancer genes curated for canonical signaling pathways reported in Sanchez-Vega^19^ which are altered recurrently in tumor samples. We choose to focus on this gene set as genetic alterations beyond these genes are either rare or the functional impact is unclear. The gene expression data matrix includes upper quartile normalized RSEM data^1^ from mRNA-seq for 1239 genes, the union of 1000 most variable genes from the transcriptome (capturing the dominant variation in the dataset which is the cell-of-origin as reported in Hoadley et al.^1^) plus 141 immune-related genes and 103 stemness marker genes which will be described shortly. This dataset is assembled this way to reflect the fact that the molecular phenotype of a tumor is complex and influenced by a multitude of factors including cell-of-origin, histology (e.g., squamous vs. adenocarcinoma), tumor microenvironment (e.g., immune cell infiltration), dedifferentiation states, and oncogenic pathway activation. It allows a direct comparison of the Nebula analysis with the existing unsupervised integrative clustering approach.

To construct the biological graph, ten canonical oncogenic pathways are considered: cell cycle, Hippo, Myc, Notch, oxidative stress response/Nrf2, PI-3-Kinase/Akt, receptor-tyrosine kinase/RTK-RAS, TGF$\beta$ signaling, p53 and $\beta$-catenin/Wnt signaling, which are frequently altered at the genetic and epigenetic level, focusing on pathway members likely to be cancer drivers or therapeutic targets^19^. An edge is drawn between two genes if they belong in the same oncogenic pathway. Members of these pathways and their interactions have been captured in a number of pathway databases, such as Pathway Commons^20^, REACTOME^21^ and KEGG^22^.

At the mRNA expression level, we included the immune marker set as defined in Yoshihara et al., (2013)^23^ and a stemness index defined in Malta et al.^24^. Immune cell infiltration characterizes tumor microenvironment and can be quantified by the immune pathway gene expressions using RNA-seq data. It has been shown as a good prognostic index in several tumor types and can inform the design of immunotherapy treatment strategies^25,26^. The stemness index measures oncogenic dedifferentiation from the cell of origin and acquisition of progenitor-like, stem-cell-like features. It has been associated with distant metastasis, disease progression and poor prognosis. The signaling networks and pathways are converted to undirected graphs with vertices representing genes in the networks and edges corresponding to biological interactions within and between modalities.

We ran Nebula across a set of parameter settings ($\alpha _0 \in \{10,100,1000\}$, $\eta \in$ $\{10,100,1000\}\times \{10,100,1000\}$; $\nu \in \{1,10\} \times \{1,10\} \times \{1,10\}$). From each output, we computed F-statistics for leukocyte fraction and stemness index measured by the transcriptomic changes, and $\chi ^2$ statistics for each of the 10 oncogenic signaling pathways altered at genomic and epigenomic levels. We then used Fisher’s method to combine the *p* values for these tests to select the optimal Nebula solution that show maximal differences across the biological pathway activities. We note this model selection procedure can be extended to optimize other metrics of interest such as survival difference across the subgroups which we describe later in the analysis of melanoma patient stratification.

#### Nebula identifies cancer subtypes driven by shared pathway activities across tissue sites

**Figure 2:**
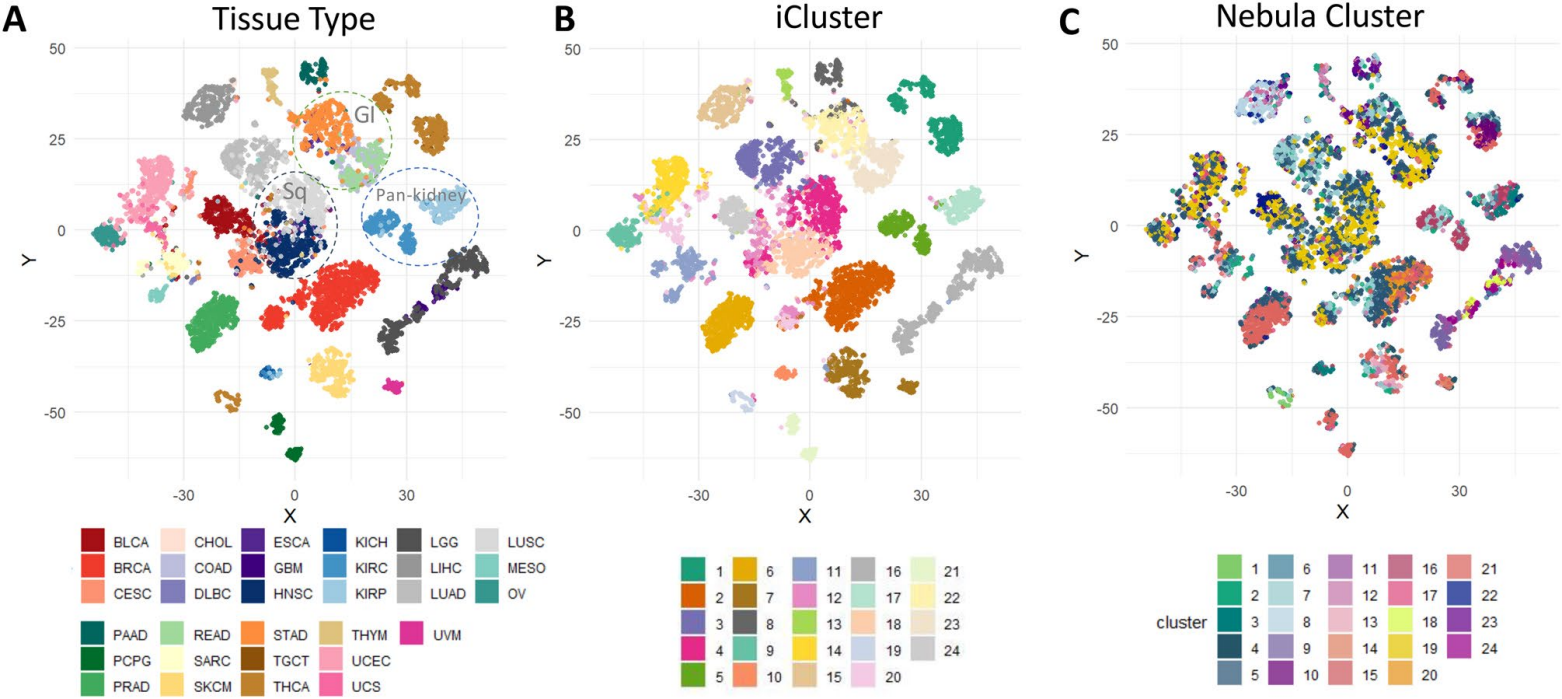
Nebula analysis identifies cancer subtypes driven by shared pathway activities across tissue sites. t-SNE plot of the TCGA  9000 tumor samples across 31 tissue sites. Each dot is a tumor sample. (**A**) Major molecular variation separates organ site where the tumor arise. Circle highlight three broader category including GI (gastrointestinal) track, Sq (squamous cell carcinomas), and Pan-kidney; (**B**) Integrative clustering on most variable genes using the iCluster algorithm; (**C**) Nebula cluster membership superimposed on the t-SNE plot.

Recent pan-cancer analyses and single cell studies have shown that conventional unsupervised clustering applied to a large patient cohort is predominantly driven by major factors such as cell-of-origin or histology^1–3^. Using the t-Distributed Stochastic Neighbor Embedding (t-SNE)^27^ approach to project the data onto a two-dimensional map clearly shows that tissue site explains the major variation in the data (Fig. 2A), which is consistent with the TCGA pan-cancer cell-of-origin study^1^ as well as in single cell populations^2,3^. Tumors from the same organ site cluster tightly around each other. Cancers from sites anatomically connected (e.g., GI track including esophageal (ESCA), stomach (STAD), colorectal (COAD, READ)), or of the same histology (e.g., squamous cell carcinomas including lung (LUSC) and head and neck (HNSC)) are further positioned close to each other in molecular distances (Fig. 2A). This is expected with unsupervised clustering performed on most variable feature which captures cell-of-origin as the dominant variation. Indeed, when we applied an integrative clustering analysis on the most variable features using the iCluster algorithm^14^, we obtained clusters primarily driven by organ sites (Fig. 2B).

**Figure 3:**
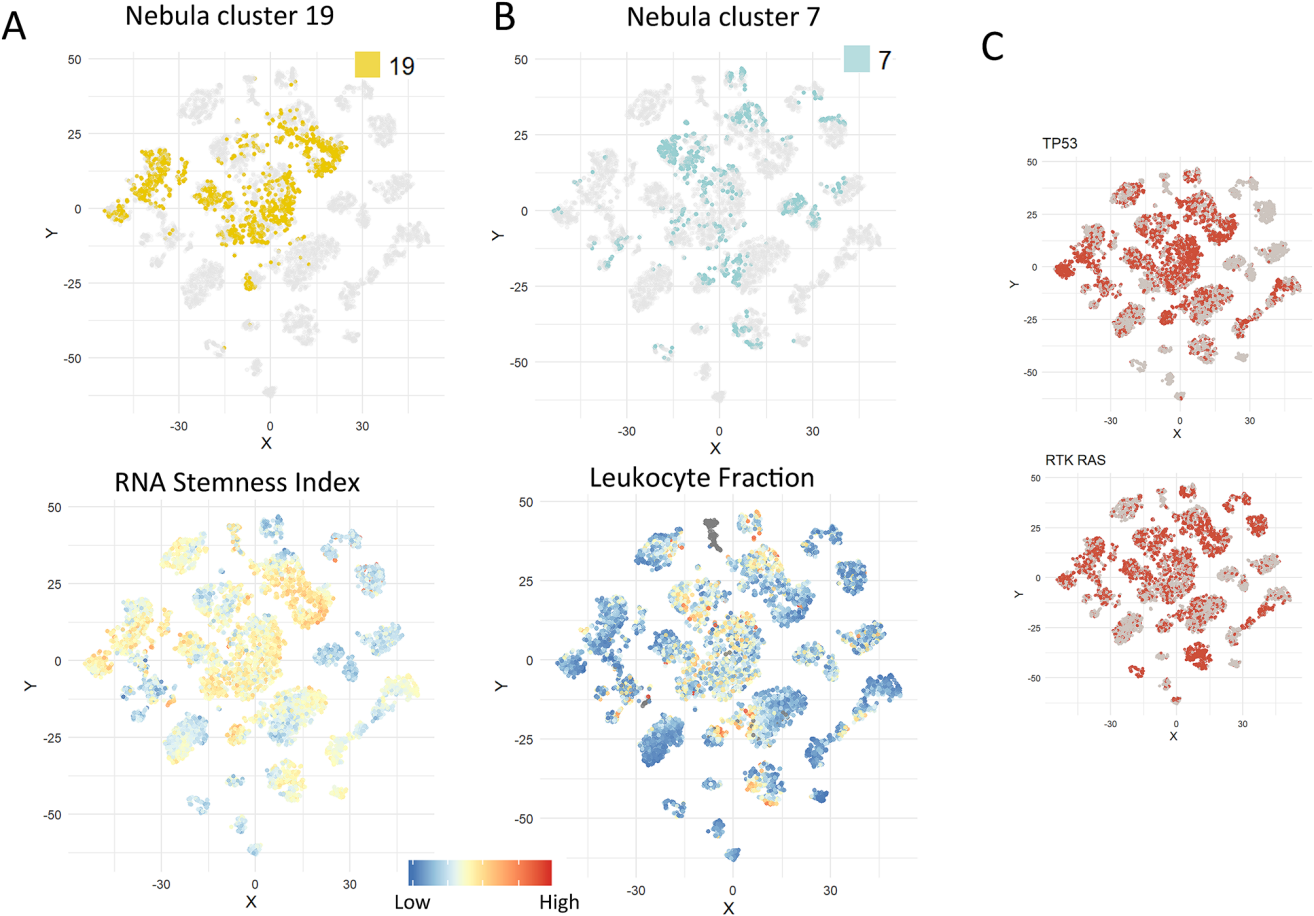
Highlighting Nebula clusters driven by stemness phenotype and immune marker activities, TP53 and RTK-RAS pathway activities.

If identifying clusters with shared biology rather than tissue type is of interest, we show Nebula provides a more directed approach. In fact, the majority of Nebula clusters are highly mixed in tissue type (Supplementary Fig. 1A) and formed by shared oncogenic signaling and pathway activities with potential therapeutic implications (Fig. 2C, Supplementary Figs. 1 and 2). For example, cluster 19 (yellow) is driven by high stemness index (Fig. 3A). This cluster included a total 1,366 individual tumors across 12 tumor types including subsets of bladder, cervical, colorectal, head and neck, lung squamous and endometrial cancer. Most samples in this cluster are also enriched for TP53 and RTK-RAS pathway alterations (Fig. 3C). Cluster 7 (light blue) is characterized by high leukocyte fraction (immune infiltration) (Fig. 3B), with a total of 915 tumors across multiple cancer types including melanoma, lung adenocarcinomas, breast and kidney cancer. A few disease types remain strongly distinct in this analysis including gliomas (GBM and LGG) which form the tissue-type dominant clusters 9, 11, 18 and 24, pan-kidney (cluster 16), and thyroid cancer (clusters 1 and 10) (Supplementary Fig. 1A).

Nebular incorporates a feature selection process in the Dirichlet process mixture model by using a feature and patient specific indicator to infer cluster-active vs cluster-inactive states with different priors (see “Methods” Section). Biological and regulatory networks are embedded into indirect graphs with a multi-modal Ising model to induce coupling effect among selection status of connected genes. Supplementary Fig. 3 shows the mRNA expression features selected in the TCGA pan-cancer analysis. All of the immune and stemness markers were selected together across all of the 24 clusters as we enforced a strong prior on these pathways, while only a small fraction of the most variable genes were selected reducing the effect from lineage-specific differences across cancer sites.

#### Nebula identifies melanoma subtypes with distinct clinical outcome

**Figure 4:**
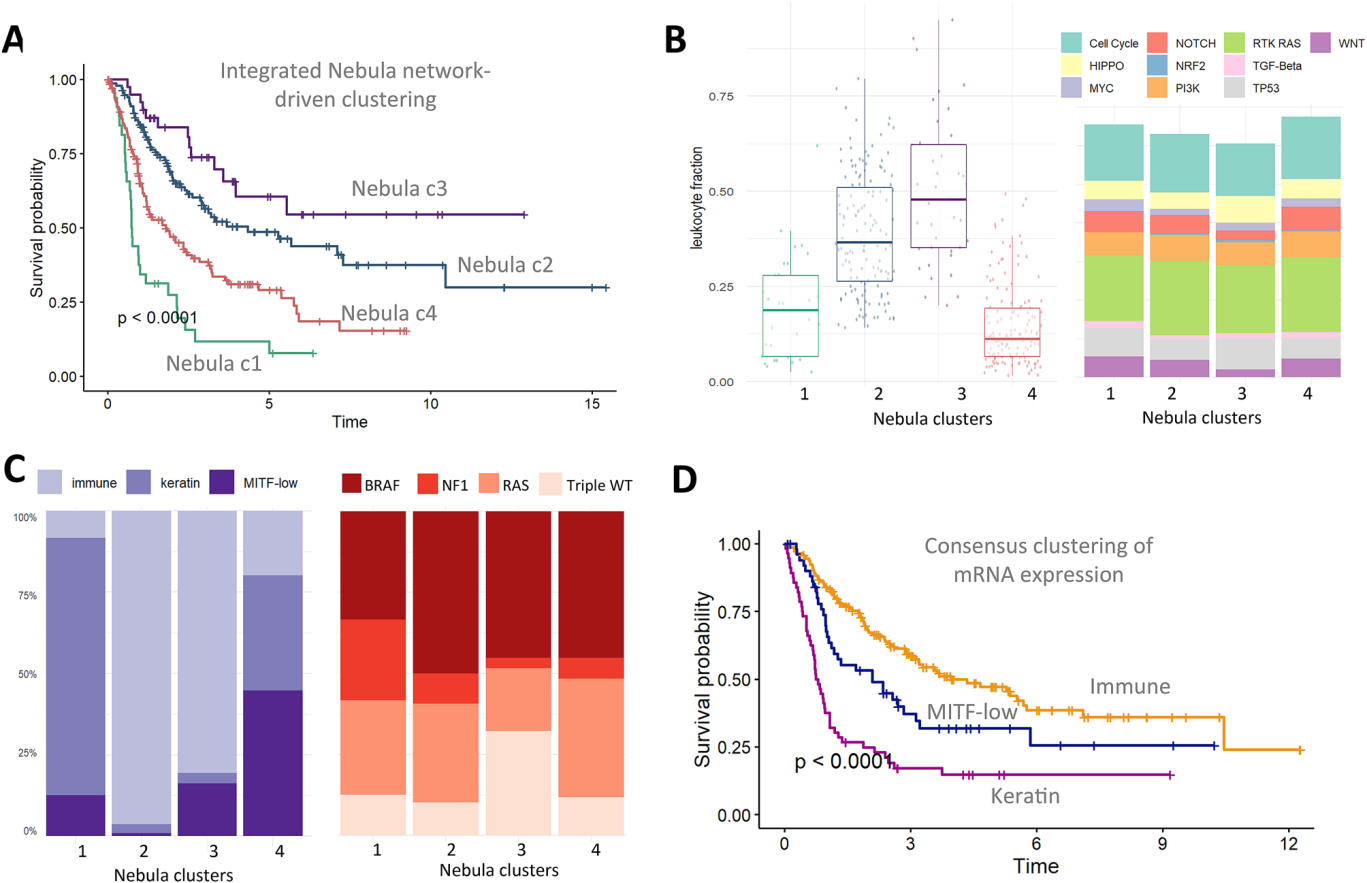
Integrated network-based clustering identifies four melanoma subtypes. (**A**) Kaplan–Meier survival curves for the Nebula clusters; (**B**) Leukocyte fraction and DNA alteration of the 10 oncogenic pathways across the Nebula clusters; (**C**) Comparison of the Nebula clusters to the TCGA mRNA expression and mutation subtypes; (**D**) Kaplan–Meier survival curves for the TCGA immune subtype.

The clinical role of tumor-infiltrating lymphocytes has been established in several cancer types^25,26,28^. Here we perform a Nebula analysis using the same biological graphs as used in the full pan-cancer analysis on the 363 TCGA melanoma samples and eventually identify 4 subtypes with distinct prognostic outcomes, Clusters 2 and 3 show higher lymphocyte fraction and association with good prognosis (Fig. 4A,B). Cluster 3 has higher fraction of TP53 pathway alterations (Fig. 4B) and higher fraction of triple-WT tumors (wild-type for *BRAF, NRAS,* and *NF1*) (Fig. 4C). The 5-year survival rate for Nebula identified immune-associated cluster 2 and 3 are 49% (95%CI: 39–60%) and 61% (95%CI: 45–82%) respectively, significantly better than cluster 1 and 4 with a 5-year survival rate of 8% (95%CI: 2–29%) and 29% (95%CI: 21–40%). The Nebula cluster stratification in survival outcome (Fig. 4A) is also more striking than TCGA mRNA expression subtypes (Fig. 4D) derived from consensus hierarchical clustering^28^, with a 5-year survival rate of 47% (immune subtype), 32% (MITF-low) and 15% (Keratin subtype). Here we show that by incorporating biological knowledge as prior, Nebula can enhance patient stratification toward identifying clinically relevant subtypes.

### Immune cell decomposition from single cell data using Nebula

**Figure 5:**
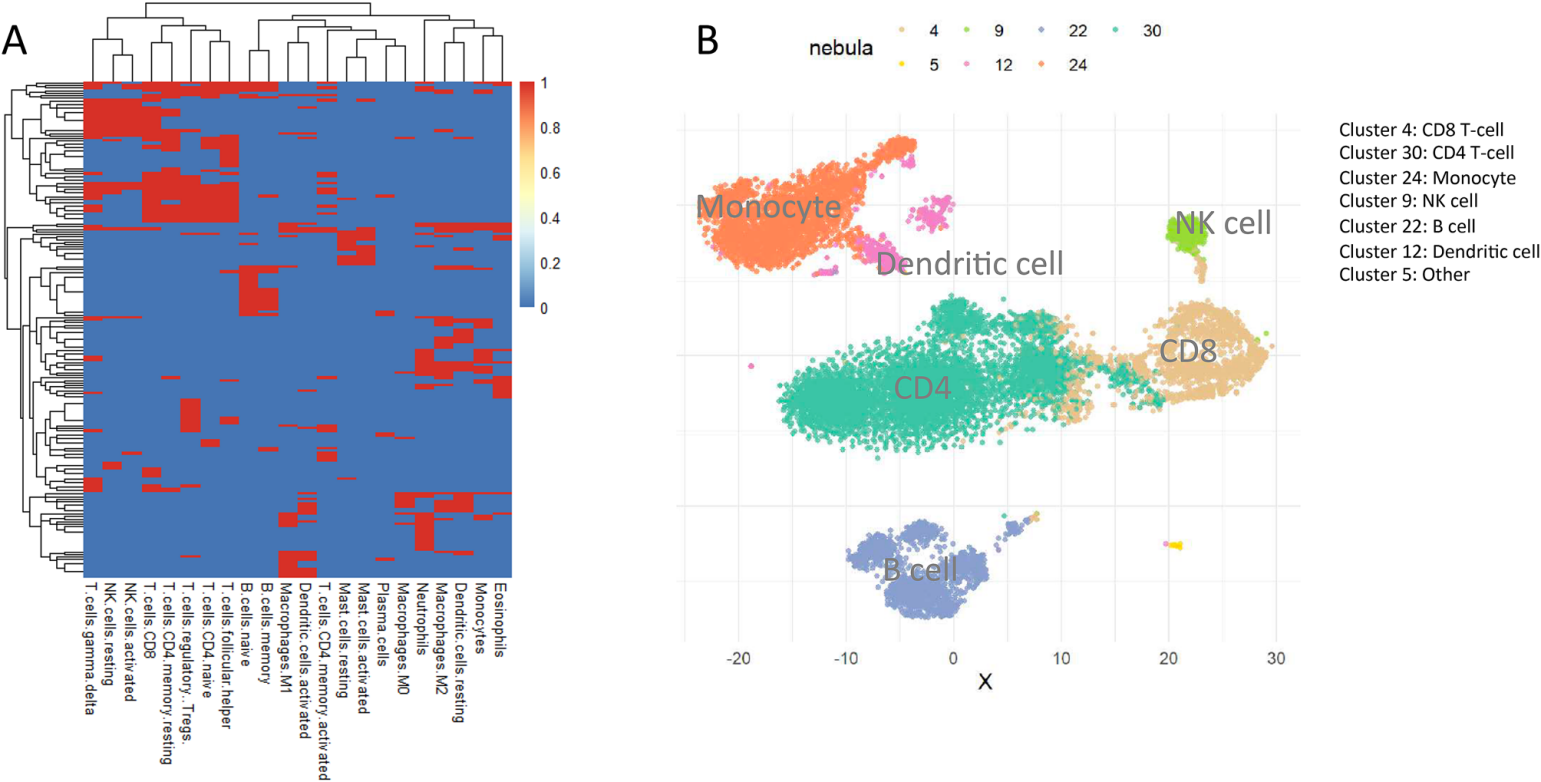
Nebula analysis of single cell RNA-seq data of peripheral blood mononuclear cells (PBMCs). (**A**) Leukocyte signature matrix of 22 functionally defined immune gene sets (column) across 547 genes (row) that distinguish hematopoietic cell phenotypes as described in Newman et al. The heatmap matrix indicates whether a gene has standardized reference expression above 0.1 (red) in the corresponding cell type as an additional filtering step for the immune subsets associated markers as Nebula network input. (**B**) t-SNE plot of the 12,039 PBMCs with color indicating Nebula cluster assignments.

Finally, we describe Nebula analysis of a single-cell RNA-seq dataset^29^ profiling 68k peripheral blood mononuclear cells (PBMCs) from a healthy donor to dissect immune cell populations. Our analyses include a total of 12,039 cells after filtering as in Cole et al.^30^ and data pre-processing as described in Lopez et al.^31^. To map cell subpopulations, state-of-the-art single cell pipelines (e.g., Seurat) typically use k-means or hierarchical clustering of the most variable genes^8,29^. Nebula offers several advantages over deterministic algorithms through a Bayesian probabilistic inference framework and the selection of features that characterize each subpopulation. In addition, we incorporated prior information in graph structures on 22 functionally defined human immune subsets across 547 genes that distinguish hematopoietic cell phenotypes as describe in Newman et al.^32^. We further restricted to marker genes with standardized reference expression value $>0.1$ for constructing the graph sets as Nebula input (Fig. 5A). This will allow more robust mapping of immune cell subpopulations against noise and unknown variabilities and allow more refined discrimination of rare or related cell types. Fig. 5B shows that Nebula maps the major different immune cell subpopulations differentiating CD4, CD8, B cells, and monocytes, as well as smaller cell subsets including NK cells and dendritic cells with high precision. The Nebula cell type assignment has a concordance rand index of 0.923 with the Seurat assignment.

## Discussion

In this paper we present Nebula, a powerful clustering approach for high-dimensional multi-modal molecular data. The method constructs a unified framework to identify subgroups along with their associated molecular features under the guidance of biological networks within and across different data types. To facilitate computational efficiency. we also develop a variational Bayes algorithm to achieve scalable implementation in high-dimensional settings. We apply the method to both pan-cancer and a single cell data. In pan-cancer, using a set of canonical oncogenic signaling pathways, an immune and a stemness index across point mutations, copy number alterations, DNA methylation and mRNA expression data we show that current clustering methods performed on most variable features inevitably lead to subgroups primarily driven by cell-of-origin. By contrast, Nebula allows targeted identification of subgroups driven by shared pathway activities of interest, and can be readily extended to include any number of additional pathways and genesets of interest for discovering shared biology across cancer types.

For the single cell data, using a scRNA data of peripheral blood mononuclear cells from a single healthy donor, we show as a proof-of-principal that Nebula can be readily applied to deconvolute heterogeneous cell subpopulations in single cell data. In this analysis, we demonstrate that Nebula can accurately identify major immune cell subsets. Unsupervised clustering performs equally well in this setting, but will be considerably less effective for mixtures with more complicated compositions with content unrelated to immune phenotypes and noise, and for discriminating closely related cell types (e.g., naive vs. memory B cells). It is anticipated that Nebula has the potential to dissect the signals of interest with more precision. Furthermore, it can be applied to multi-modal single cell data to dissect heterogeneous cell populations. The Nebula method and software tool we developed here will speed up exciting biological discoveries potentially relevant to clinical applications with the increasing amount of multi-model datasets generated from patient samples in both bulk tumor and single cell settings.

## Methods

### Nonparametric clustering embedded with feature selection

We formulate the model for general multi-modal data structure. Suppose there are *n* subjects with *M* data modalities generated from different sources, e.g. biological procedures including gene expression, DNA alternation, etc. We denote the overall observed data as ${\mathbf {X}}=({\mathbf {X}}^{(1)},\dots ,{\mathbf {X}}^{(M)})=({\mathbf {x}}^T_1,\dots ,{\mathbf {x}}^T_n)^T$ with $n\times p_m$ matrix ${\mathbf {X}}^{(m)}=\{x^{(m)}_{ij}\}$ representing $p_m$ features within data modality *m* across all the subjects, and ${\mathbf {x}}_i$ summarizing observations over $p=\sum _{m}p_m$ features for subject *i*. The collected features are dependent with each other within and between each data modality. For example, the pathway membership within gene expression data, the regulation between gene expression and DNA alternation. Such information essentially can be transformed into undirected graphs with nodes representing features and edges indicating the existence of their relationship. Here, we use graph $\mathcal {G}^{(m)}$ to capture the structrual information within modality *m*, and $\mathcal {G}^{(m,g)}$ for the relationship between modalities *m* and *g*.

To study data structure and subject similarity in the presence of high dimensional data, clustering embedded with feature selection should be performed to group observations with respect to the identified features that define each group. However, there is a lack of unified approach to achieve so. To conduct clustering while simultaneously distinguish the contribution of features, we introduce a dummy indicator $\gamma ^{(m)}_{ij}\in \{0, 1\}$ to denote the selection status of feature *j* within modality *m* for subject *i*. Vector $\varvec{\gamma }^{(m)}_i=(\gamma ^{(m)}_{i1},\dots ,\gamma ^{(m)}_{ip_m})^T$ partitions each observation from modality *m* into informative and non-informative components. Accordingly, the informative piece is modeled under parameter set $\Theta ^{(m)}_{i1}=(\varvec{\theta }^{(m)}_{i11},\dots ,\varvec{\theta }^{(m)}_{i1p_m})$, which varies by subject index to distinguish the definition for each cluster; and the noninformative set is associated with parameter set $\Theta ^{(m)}_{0}=(\varvec{\theta }^{(m)}_{01},\dots ,\varvec{\theta }^{(m)}_{0p_m})$, which is subject independent indicating minimal observation variation. We carry out the joint analyses under the following Bayesian nonparametric model

1 $$\begin{aligned} \begin{aligned} {\mathbf {x}}_i\mid \{ \varvec{\gamma }^{(m)}_i, \Theta ^{(m)}_{i1}, \Theta ^{(m)}_{0}\}^M_{m=1}&\sim F(\{ \varvec{\gamma }^{(m)}_i, \Theta ^{(m)}_{i1}, \Theta ^{(m)}_{0}\}^M_{m=1}),\\ \{ \varvec{\gamma }^{(m)}_i, \Theta ^{(m)}_{i1}, \Theta ^{(m)}_{0}\}^M_{m=1}\mid G&\sim G,\\ G&\sim DP(G_0, \alpha _0 ), \end{aligned} \end{aligned}$$

with $i=1,\dots ,n$. Specifically, $F(\cdot )$ is the conditional distribution over modalities and features for ${\mathbf {x}}_i$, which can be represented as a product of distribution function in each modality $f_m, m=1,\dots ,M$. Depending on the data generation process, $f_m$ varies among different distributions e.g. Normal, Bernoulli, Poisson, etc. In other words, each observation is generated from a two-component mixture

2 $$\begin{aligned} x^{(m)}_{ij}\mid \gamma ^{(m)}_{ij}, \varvec{\theta }^{(m)}_{i1j}, \varvec{\theta }^{(m)}_{0j} \sim \gamma _{ij}^{(m)}f_{m}(\varvec{\theta }^{(m)}_{i1j})+(1-\gamma ^{(m)}_{ij})f_{m}(\varvec{\theta }^{(m)}_{0j}), \end{aligned}$$

where the joint distribution of unknown parameters follows function *G* and we assign *G* to be a nonparametric Dirichlet process (DP) distribution with base measure $G_0$ and concentration parameter $\alpha _0$. Here $G_0$ provides prior support for the samples to center on, and $\alpha _0$ controls the strength of concentration. In order to conduct posterior inference, we rewrite model (1) by a stick-breaking representation based on weighted sums of infinite point masses as

3 $$\begin{aligned} \begin{aligned} G&=\sum _{h=1}^\infty w_h' \delta _{{\mathbf {c}}_h}; \\ w_h'&= w_h \prod _{l=1}^{h-1}(1-w_l); \quad&w_l \sim \text {Beta}(1, \alpha _0); \quad h=1,\dots ,\infty , \end{aligned} \end{aligned}$$

where $\delta _{{\mathbf {c}}_h}$ is the point mass at ${\mathbf {c}}_h$, with ${\mathbf {c}}_h\sim G_0$. Function *G* is an infinite discrete distribution, and realizations of $\{ \varvec{\gamma }^{(m)}_i, \Theta ^{(m)}_{i1}, \Theta ^{(m)}_{0}\}^M_{m=1}$ can be obtained by sampling from random draws $\{{\mathbf {c}}_h\}_{h=1}^{\infty }$ under weight $\{w_h'\}_{h=1}^{\infty }$. Eventually, the discrete nature of DP allows to cluster subjects with identical set of parameter values. The informative features defining each cluster will be captured by the selection status, and the estimated cluster specific distributions will be crucial for future subject classification.

### Biological plausible priors

We assign priors for the unknown parameters in models (1), (3). In the TCGA data, as described above, we integrate gene expression and DNA alteration for each patient. Therefore, distribution (2) for $x^{(m)}_{ij}, i=1,\dots ,n, j=1,\dots ,p_m, m=1,2$ becomes

4 $$\begin{aligned} \begin{aligned} x^{(1)}_{ij}\mid z_i, \gamma _{ij}^{(1)}, \mu _{ij}, \mu _{0j}, \sigma ^{2}_{ij}, \sigma ^{2}_{0j}&\sim \gamma ^{(1)}_{z_ij}\text{ N }(\mu _{z_ij},\sigma ^{2}_{z_ij})+(1-\gamma ^{(1)}_{z_ij})\text{ N }(\mu _{0j},\sigma ^{2}_{0j});\\ x^{(2)}_{ij}\mid z_i, \gamma _{ij}^{(2)}, p_{ij}, p_{0j}&\sim \gamma ^{(2)}_{z_ij}\text{ Bern }(p_{z_ij})+(1-\gamma ^{(2)}_{z_ij})\text{ Bern }(p_{0j}). \end{aligned} \end{aligned}$$

Based on (4), we assume the partial base measure related to the joint distribution of $(\mu _{ij}, \sigma ^{2}_{ij})$ to be normal-inverse-gamma, i.e. $\text{ N-IG }(0, \lambda , \alpha _\sigma , \beta _\sigma )$ and that related to the distribution of $p_{ij}$ as $\text {Beta}(\alpha _p,\beta _p)$, to induce discrimination among clusters. In terms of the parameters for the noice features, we directly set point mass 0 and 20 as the partial base measure for $\mu _{0j}$ and $\sigma ^{2}_{0j}$ to introduce a flat, noninformative prior support for the noninformative features that contribute to none of the clusters. Similarly, we set the base measure for the distribution of $p_{0j}$ to be point mass at 0.5. The prior specification in the single cell data analysis directly follows the above setting.

The underlying molecular dependency within and cross modalities is often omitted in existing clustering analysis, but provides crucial biological guidance to define patient subtypes. Here, based on the extract molecular network information, we assume the biologically graph within gene expression as $\mathcal {G}^{(1)}$, that within DNA alternation as $\mathcal {G}^{(2)}$, and within two modalities as $\mathcal {G}^{(1,2)}$. We modify Ising model to accommodate different modality components and impose the following prior for indicator $\{\varvec{\gamma }^{(1)}, \varvec{\gamma }^{(2)}\}$

5 $$\begin{aligned} \begin{aligned} \pi (\varvec{\gamma }_i^{(1)}, \varvec{\gamma }_i^{(2)})&\propto \exp \left( -\sum _{m=1}^2 \eta _m \sum _{j=1}^{p_m} \gamma ^{(m)}_{ij} + \sum _{m=1}^2 \sum _{ j \sim _{\mathcal {G}^{(m)}} k} \nu _m I[\gamma ^{(m)}_{ij}=\gamma ^{(m)}_{ik}] \right. \\&\qquad \left. + \sum _{j \sim _{\mathcal {G}^{(1,2)}} k} \nu ' I[\gamma ^{(1)}_{ij}=\gamma ^{(2)}_{ik}] \right) , \end{aligned} \end{aligned}$$

where $j \sim _\mathcal {G}k$ indicates features *j* and *k* are adjacent in graph $\mathcal {G}$. Model (5) induces a coupling effect over biological networks with $\eta _1$ and $\eta _2$ adjusting the sparsity of informative gene and DNA biomarkers, $\nu _1$, $\nu _2$ and $\nu '$ controlling the smoothness over network in each modality and between them. By incorporating prior (5) into our joint clustering framework, we can enhance both grouping and feature identification under guidance from underlying biologically networks, which may provide more meaningful biologically interpretation compared with existing alternatives.

### Variational Inference

We develop posterior inference algorithms to estimate model parameters for Nebula. To estimate posterior distributions, we develop the Markov Chain Monte Carlo (MCMC) algorithm via Gibbs sampler by drawing from the conditional probability distribution of each parameter. However, due to the high dimensional feature space, MCMC algorithms are computationally intensive and difficult to scale up in real practice. Therefore, we develop a variational Bayes (VB) inference^33^ algorithm, which approximates the posterior distribution by a simpler distribution, called the variational distribution. We have done extensive numerical studies to test the performance of both algorithms, and observed the VB algorithm reduces computational cost for around 100 folds compared to the MCMC approach. We describe below the construction details for the VB algorithm.

To fix the ideas, note that the model parameters are now $\Xi = ({\mathbf {z}},{\mathbf {w}},\gamma ,\mu ,\sigma ^2,P)$ where ${\mathbf {z}}$ is an *n*-dimensional vector, ${\mathbf {w}}$ is an $\infty$-dimensional vector, $\gamma$ is a $p \times \infty$ matrix, $\mu$ and $\sigma ^2$ are $p_1 \times \infty$ matrices, and *P* is an $p_2 \times \infty$ matrices. For notational convenience, we keep $\mu$, $\sigma ^2$, and *P* as $p \times \infty$ matrices where the lower $p_2$ rows of $\mu$, $\sigma ^2$ and the upper $p_1$ rows of *P* are unused. The posterior loglikelihood deduced from the model is given in Supplementary Note.

In variational inference, the variational distribution is often assigned a product (independent) measure of the individual model parameters, as it facilitates computation and leads to scalability. Thus, we take a product measure on ${\mathbf {z}}$, ${\mathbf {w}}$, $\gamma$, $(\mu ,\sigma ^2)$, and *P* for the variational measure $q(\Xi )$ as follows.

$$\begin{aligned} q({\mathbf {z}},{\mathbf {w}},\gamma ,\mu ,\sigma ^2,P) = q({\mathbf {z}}) q({\mathbf {w}}) q(\gamma ) q(\mu ,\sigma ^2) q(P), \end{aligned}$$

where under *q*,

$$\begin{aligned} z_i&\sim \text {Multi} \left( \frac{e^{b_{i1}}}{\sum _l e^{b_{il}}}, \dots , \frac{e^{b_{iH}}}{\sum _l e^{b_{il}}} \right) ,\\ w_h&\sim \text {Beta}(f_h,g_h), \quad 1 \le h < H,\\ \gamma _{jh}&\sim \text {Bern}(\mathrm {expit}(c_{jh})), \quad 1\le j \le p, 1 \le h \le H,\\ \sigma _{jh}^2&\sim \text {IG}(d_{jh}/2,r_{jh}/2), \quad 1 \le j \le p_1, 1 \le h \le H,\\ \mu _{jh}|\sigma _{jh}^2&\sim \text {N}(m_{jh},\sigma _{jh}^2/v_{jh}), \quad 1 \le j \le p_1, 1 \le h \le H,\\ p_{jh}&\sim \text {Beta}(s_{jh},t_{jh}), \quad p_1+1 \le j \le p, 1 \le h \le H, \end{aligned}$$

where $\mathrm {expit}(c) = 1/(1+e^{-c})$. Note that the variational distribution restricts the number of clusters *H* by setting $w_H=1$ with probability 1 for some *H*, although our model does not restrict the number of clusters.

### Algorithm

We approximate the posterior distribution by maximizing the evidence lower bound (ELBO), which is defined as

6 $$\begin{aligned} \text {ELBO} = \mathbb {E}_q \log \pi (\Xi ,X) - \mathbb {E}_q \log q(\Xi ), \end{aligned}$$

It is straightforward to see that

$$\begin{aligned} \log \pi (X) - \text {ELBO} = D_\text {KL}(q(\Xi )||\pi (\Xi |X)), \end{aligned}$$

where the RHS refers to the Kullback–Leibler divergence between the posterior distribution and the variational measure, which is always *nonnegative*. This implies (1) maximizing ELBO is equivalent to minimizing the Kullback–Leibler divergence and therefore it is fair to say that the posterior distribution is approximated by the variational measure and (2) ELBO being maximized gets close to the evidence $\log \pi (X)$.

The formula of ELBO is analytically available and we optimize ELBO by the (blockwise) coordinate descent algorithm. The complete algorithm for the Nebula under VB is presented in online Appendix. It is worth noting the time complexity of the algorithm is $\mathcal {O}(npH+eH)$ per iteration where *e* is the number of edges in the graphs $\mathcal {G}^{(1)}$, $\mathcal {G}^{(2)}$, and $\mathcal {G}^{(1,2)}$, which is extremely efficient for any practical use.

### Model selection and evaluation

To implement the Nebula, we will need to pre-specify hyper-parameter values for $\alpha _0$, $\varvec{\eta }=(\eta _1,\eta _2)$ and $\varvec{\nu }=(\nu _1,\nu _2,\nu ')$. Of note, compared with parametric clustering methods, the Nebula does not require to pre-determine the number of clusters. During the model implementation, we can always choose a conservative value for *H* to make it much larger than the cluster number.

In the analyses, our goal is to achieve clustering with biological variability across the pathways we are interested in. To find the most desirable solution, We run the Nebula under a range of parameter values including $\alpha _0 \in \{10, 100, 1000\}$, $\varvec{\eta }\in \{10, 100, 1000\} \times \{10, 100, 1000\}$, and $\varvec{\nu }\in \{1,10\} \times \{1,10\} \times \{1,10\}$. From each output, we compute the *F*-statistics for the immune and stemness index measured by the transcriptomic changes in the TCGA pan-cancer analysis, and the $\chi ^2$ statistics for each of the 10 oncogenic signaling pathways alter at genomic and epigenomic levels. We then use the Fisher’s method to combine the *p* values for these tests to select the optimal solution that explains maximal differences across the biological pathway activities. In the single cell data analysis, we used a similar strategy to compute an integrated F statistic across the CIBERSORT^32^ immune marker subsets. In a homogeneous cohort setting, other calculations may be appropriate to refine parameter selection and evaluate results. For example, calculating log-rank statistic to maximize survival differences between groups.

## Supplementary information

Supplementary Figures.

Supplementary Information 1.

Supplementary Information 2.

## Data Availability

The two data sets used in this study are publicly available. The TCGA data set is available at https://portal.gdc.cancer.gov/. The single cell RNAseq data set is available from Zheng et al.^29^.

## Code availability

A software implementation of Nebula is available in the public domain at https://github.com/nebula-group/nebula. Instruction on installation and a tutorial on running Nebula analysis is also provided.
