# Supplementary material for: Bayesian network-driven clustering analysis with feature selection for high-dimensional multi-modal molecular data
Source: Sci Rep. 2021 Mar 4;11:5146. doi: 10.1038/s41598-021-84514-0 (PMC7933297; doi:10.1038/s41598-021-84514-0)
Supplement: Supplementary file 1 — Supplementary Figures. [file 41598_2021_84514_MOESM1_ESM.pdf]

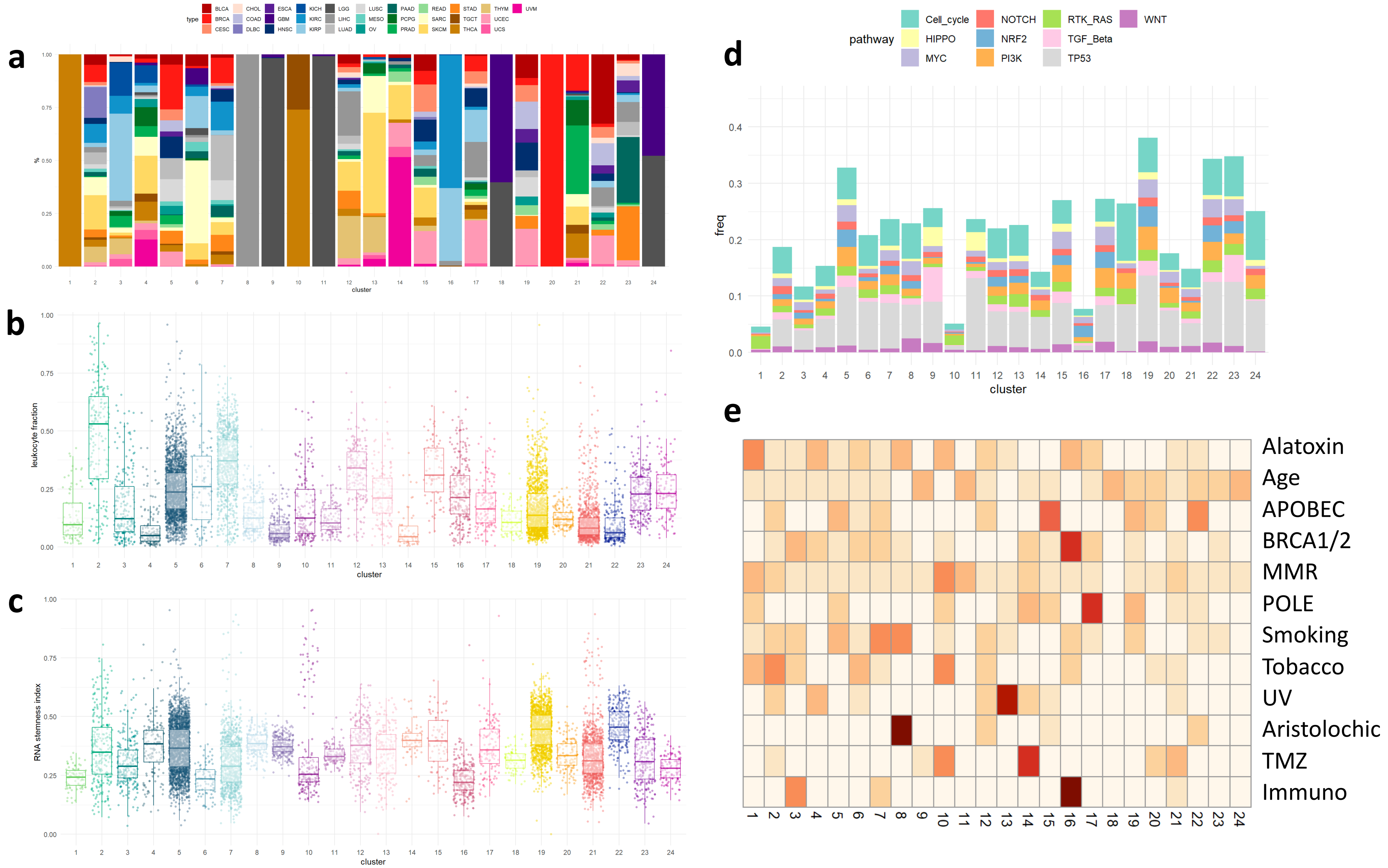

Supplementary Figure 1. Pan-cancer integrated network-based clustering identifies subgroups driven by shared pathway activity. **a**. Tissue type composition in Nebula clusters. **b**. Leukocyte fraction by each cluster. c. Stemness index by each cluster. **d**. Frequency of alteration at the oncogenic signaling pathway level. e**.** Mutational signature by each clster.

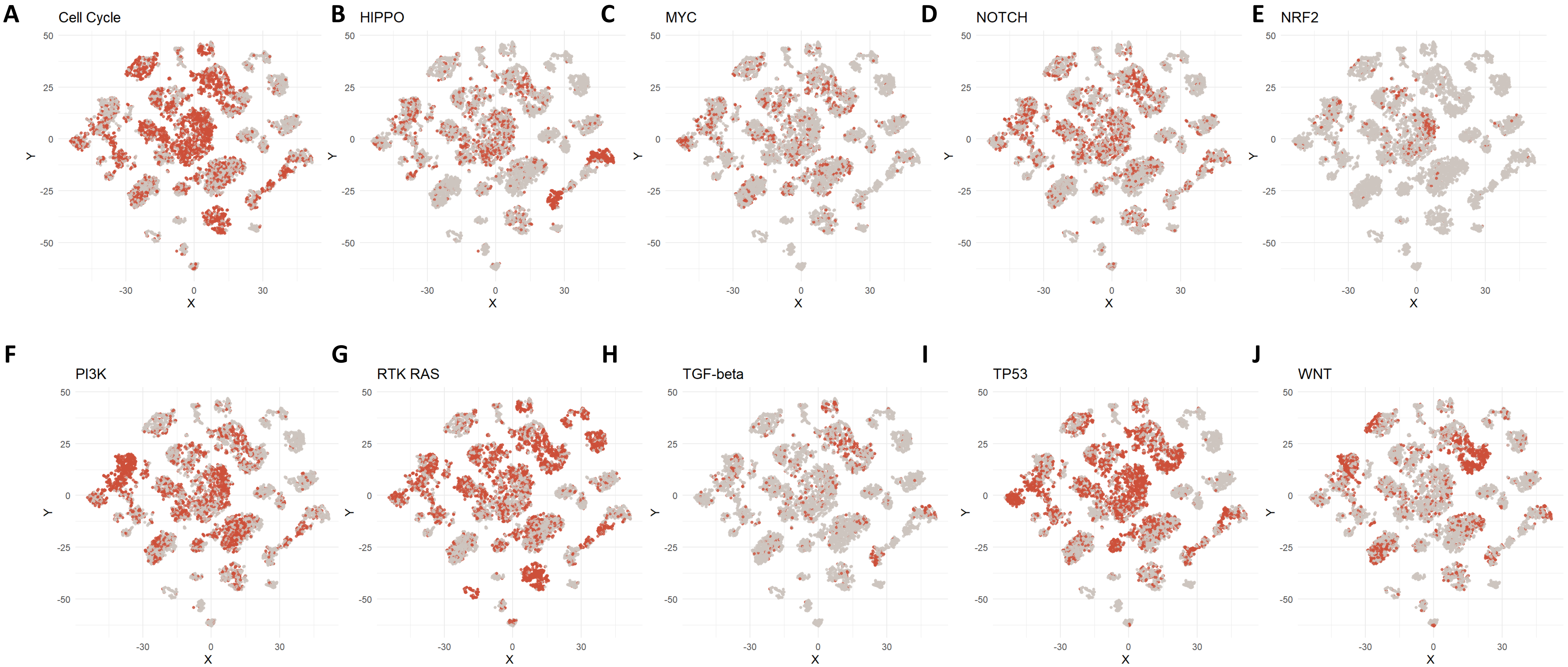

Supplementary Figure 2. t-SNE plot superimposing oncogenic signaling pathway alterations.

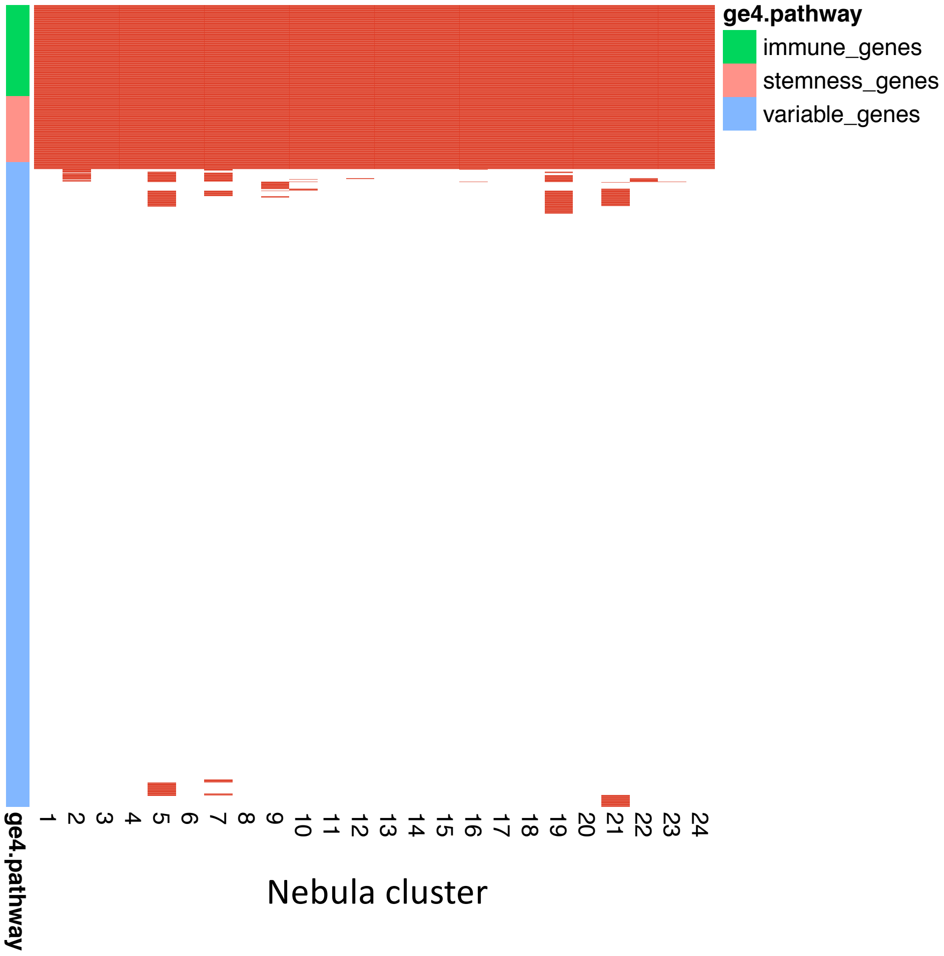

Supplementary Figure 3. Feature selection performance on the TCGA pan-cancer data analysis showing the selection of the immune and stemness markers universally across the Nebula clusters as we enforced a strong prior on these pathways, while only a small fraction of the most variable genes were selected reducing the effect from lineage-specific differences across cancer sites.
