# Supplementary material for: Bayesian network-driven clustering analysis with feature selection for high-dimensional multi-modal molecular data
Source: Sci Rep. 2021 Mar 4;11:5146. doi: 10.1038/s41598-021-84514-0 (PMC7933297; doi:10.1038/s41598-021-84514-0)
Supplement: Supplementary file 2 — Supplementary Information 1. [file 41598_2021_84514_MOESM2_ESM.pdf]

# Bayesian Network-Driven Clustering Analysis with Feature Selection for High-Dimensional Multi-Modal Molecular Data

Yize Zhao, Changgee Chang, Margaret Hannum, Jasme Lee & Ronglai Shen

### Loglikelihood of the Model

The complete loglikelihood of our model is given by

$$
\begin{aligned}
\log \pi(\Xi|X) = C &- \frac{1}{2}\sum_{i=1}^{n}\sum_{h=1}^{\infty}\mathbb{I}(z_i = h)\sum_{j=1}^{p_1}\gamma_{jh}\left(\log\sigma_{jh}^2 + \frac{(x_{ij}-\mu_{jh})^2}{\sigma_{jh}^2}\right) \\
&- \frac{1}{2}\sum_{i=1}^{n}\sum_{h=1}^{\infty}\mathbb{I}(z_i = h)\sum_{j=1}^{p_1}(1-\gamma_{jh})\left(\log\sigma_{j0}^2 + \frac{(x_{ij}-\mu_{j0})^2}{\sigma_{j0}^2}\right) \\
&+ \sum_{i=1}^{n}\sum_{h=1}^{\infty}\mathbb{I}(z_i = h)\sum_{j=p_1+1}^{p_1+p_2}\gamma_{jh}(x_{ij}\log p_{jh} + (1-x_{ij})\log(1-p_{jh})) \\
&+ \sum_{i=1}^{n}\sum_{h=1}^{\infty}\mathbb{I}(z_i = h)\sum_{j=p_1+1}^{p_1+p_2}(1-\gamma_{jh})\left(x_{ij}\log p_{j0} + (1-x_{ij})\log(1-p_{j0})\right) \\
&+ \sum_{i=1}^{n}\sum_{h=1}^{\infty}\mathbb{I}(z_i = h)\left(\log w_h + \sum_{l=1}^{h-1}\log(1-w_l)\right) + (\alpha_0 - 1)\sum_{h=1}^{\infty}\log(1-w_h) \\
&- \eta\sum_{h=1}^{\infty}\sum_{j=1}^{p}\gamma_{jh} + \nu\sum_{h=1}^{\infty}\sum_{j<k}a_{jk}\mathbb{I}(\gamma_{jh} = \gamma_{kh}) \\
&- \frac{1}{2}\sum_{h=1}^{\infty}\sum_{j=1}^{p_1}\left(\log\sigma_{jh}^2 + \frac{\lambda\mu_{jh}^2}{\sigma_{jh}^2}\right) - (\alpha_\sigma/2+1)\sum_{h=1}^{\infty}\sum_{j=1}^{p_1}\log\sigma_{jh}^2 - \sum_{h=1}^{\infty}\sum_{j=1}^{p_1}\frac{\beta_\sigma}{2\sigma_{jh}^2} \\
&+ \sum_{h=1}^{\infty}\sum_{j=p_1+1}^{p_1+p_2}\left((\alpha_p - 1)\log p_{jh} + (\beta_p - 1)\log(1-p_{jh})\right).
\end{aligned}
$$

### Nebula Algorithm

Before we describe the algorithm, note that for $1 \le h \le H$,

$$\tau_{ih} = \mathbb{E}_q\mathbb{I}(z_i = h) = \frac{e^{b_{ih}}}{\sum_l e^{b_{il}}}.$$

For $1 \leq h < H$, we have

$$\mathbb{E}_q \log w_h = \psi(f_h) - \psi(f_h + g_h), \quad \mathbb{E}_q \log(1 - w_h) = \psi(g_h) - \psi(f_h + g_h).$$

where $\psi$ is the digamma function. For $h \geq H$, we have $w_h = 1$ and thus

$$\mathbb{E}_q \log w_h = 0, \quad \mathbb{E}_q \log(1 - w_h) = -\infty.$$

For $1 \leq j \leq p$ and $1 \leq h \leq H$, we have

$$\zeta_{jh} = \mathbb{E}_q \gamma_{jh} = \text{expit}(c_{jh}) = \frac{1}{1 + e^{-c_{jh}}}.$$

For $1 \leq j \leq p_1$ and $1 \leq h \leq H$, we have

$$\mathbb{E}_q \log \sigma_{jh}^2 = \log(r_{jh}/2) - \psi(d_{jh}/2), \quad \mathbb{E}_q \frac{(x_{ij} - \mu_{jh})^2}{\sigma_{jh}^2} = \frac{d_{jh}(x_{ij} - m_{jh})^2}{r_{jh}} + v_{jh}^{-1}.$$

For $p_1 + 1 \leq j \leq p$ and $1 \leq h \leq H$, we have

$$\mathbb{E}_q \log p_{jh} = \psi(s_{jh}) - \psi(s_{jh} + t_{jh}), \quad \mathbb{E}_q \log(1 - p_{jh}) = \psi(t_{jh}) - \psi(s_{jh} + t_{jh}).$$

The blockwise coordinate descent algorithm goes as follows.

- Update $f_h$ and $g_h$.

  For $1 \leq h < H$, optimization with respect to $f_h$ and $g_h$ goes as follows.

$$\begin{aligned} f_h &\leftarrow 1 + \sum_{i=1}^{n} \tau_{ih}, \\ g_h &\leftarrow \alpha_0 + \sum_{i=1}^{n} \mathbb{E}_q \mathbb{I}(z_i > h) = \alpha_0 + \sum_{i=1}^{n} \sum_{l=h+1}^{H} \tau_{il}. \end{aligned} \tag{1}$$

- Update $d_{jh}$, $v_{jh}$, $m_{jh}$, and $r_{jh}$.

  For each $j \in \{1, \ldots, p_1\}$ and $h \in \{1, \ldots, H\}$, optimization with respect to $d_{jh}$, $v_{jh}$, $m_{jh}$, and $r_{jh}$ goes as follows.

$$\begin{aligned} d_{jh} &\leftarrow a_\sigma + \zeta_{jh} \sum_{i=1}^{n} \tau_{ih}, \\ v_{jh} &\leftarrow \lambda + \zeta_{jh} \sum_{i=1}^{n} \tau_{ih}, \\ m_{jh} &\leftarrow v_{jh}^{-1} \zeta_{jh} \sum_{i=1}^{n} x_{ij} \tau_{ih}, \\ r_{jh} &\leftarrow \beta_\sigma - v_{jh} m_{jh}^2 + \zeta_{jh} \sum_{i=1}^{n} x_{ij}^2 \tau_{ih}. \end{aligned} \tag{2}$$

- Update $s_{jh}$ and $t_{jh}$.

  For each $j \in \{p_1+1, \ldots, p\}$ and $h \in \{1, \ldots, H\}$, optimization with respect to $s_{jh}$ and $t_{jh}$ goes as follows.

$$
\begin{aligned}
s_{jh} &\leftarrow \alpha_p + \zeta_{jh} \sum_{i=1}^{n} x_{ij}\tau_{ih}, \\
t_{jh} &\leftarrow \beta_p + \zeta_{jh} \sum_{i=1}^{n} (1-x_{ij})\tau_{ih}.
\end{aligned} \tag{3}
$$

- Update $\zeta_{jh}$.

  The KKT condition for optimizing the log likelihood with respect to $c_{jh}$ yields

$$
\begin{aligned}
c_{jh} \leftarrow & -\frac{1}{2}\mathbb{I}(j \notin \Theta_3) \sum_{i=1}^{n} \tau_{ih} \left( \log\left(\frac{r_{jh}}{2}\right) - \psi\left(\frac{d_{jh}}{2}\right) + \frac{d_{jh}(x_{ij}-m_{jh})^2}{r_{jh}} + \frac{1}{v_{jh}} \right) \\
& + \frac{1}{2}\mathbb{I}(j \notin \Theta_3) \sum_{i=1}^{n} \tau_{ih} \left( \log \sigma_{j0}^2 + \frac{(x_{ij}-\mu_{j0})^2}{\sigma_{j0}^2} \right) \\
& + \mathbb{I}(j \in \Theta_3) \sum_{i=1}^{n} \tau_{ih}(x_{ij}\psi(s_{jh}) + (1-x_{ij})\psi(t_{jh}) - \psi(s_{jh}+t_{jh})) \\
& - \mathbb{I}(j \in \Theta_3) \sum_{i=1}^{n} \tau_{ih} \left( x_{ij} \log p_{j0} + (1-x_{ij}) \log(1-p_{j0}) \right) \\
& - \eta + \nu \sum_{k \neq j} a_{jk}(2\text{expit}(c_{kh}) - 1),
\end{aligned} \tag{4}
$$

  and then $\zeta_{jh} \leftarrow \frac{1}{1+e^{-c_{jh}}}$, which is to be repeated for all $j \in \{1, \ldots, p\}$ and $h \in \{1, \ldots, H\}$ until convergence.

- Update $\tau_{ih}$. For each $i \in \{1, \ldots, n\}$ and $h \in \{1, \ldots, H\}$, optimization of log likelihood with respective to $b_{ih}$ gives

$$
\begin{aligned}
b_{ih} \leftarrow & -\frac{1}{2}\sum_{j\notin\Theta_3}\zeta_{jh}\mathbb{E}_q\left(\log\sigma_{jh}^2+\frac{(x_{ij}-\mu_{jh})^2}{\sigma_{jh}^2}\right) \\
& -\frac{1}{2}\sum_{j\notin\Theta_3}(1-\zeta_{jh})\left(\log\sigma_{j0}^2+\frac{(x_{ij}-\mu_{j0})^2}{\sigma_{j0}^2}\right) \\
& +\sum_{j\in\Theta_3}\zeta_{jh}(x_{ij}\mathbb{E}_q\log p_{jh}+(1-x_{ij})\mathbb{E}_q\log(1-p_{jh})) \qquad (5) \\
& +\sum_{j\in\Theta_3}(1-\zeta_{jh})\left(x_{ij}\log p_{j0}+(1-x_{ij})\log(1-p_{j0})\right) \\
& +\mathbb{E}_q\log w_h+\sum_{l=1}^{h-1}\mathbb{E}_q\log(1-w_l).
\end{aligned}
$$

Note that, since $b_{ih}$ are redundant for $\mathbb{E}_q\mathbb{I}(z_i=h)$, we may take

$$
\begin{aligned}
B_i &\leftarrow \max_h b_{ih}, \\
b_{ih} &\leftarrow b_{ih}-B_i, \quad \forall h, \qquad (6) \\
\tau_{ih} &\leftarrow \frac{e^{b_{ih}}}{\sum_l e^{b_{il}}},
\end{aligned}
$$

which will prevent numerical issues.

The *Nebula* algorithm is summarized below.

**Algorithm 1:** Nebula Algorithm

**input :** $X$, $\eta$, $\nu$, $\alpha_0$, $\mu_{j0}$, $\sigma_{j0}^2$, and $p_{j0}$.
**output:** $\tau_{ih}$ and $\zeta_{jh}$.

*Initialize* $\tau_{ih}(b_{ih})$ *and* $\zeta_{jh}(c_{jh})$;
**repeat**
    *update* $f_h$ *and* $g_h$ *as in Eqn* (1);
    *update* $d_{jh}$, $v_{jh}$, $m_{jh}$, *and* $r_{jh}$ *as in Eqn* (2);
    *update* $s_{jh}$ *and* $t_{jh}$ *as in Eqn* (3);
    **repeat**
        *update* $\zeta_{jh}$ *as in Eqn* (4);
    **until** *convergence*;
    *update* $\tau_{ih}$ *as in Eqns* (5) *and* (6);
**until** *convergence*;

**Data details.**

**The TCGA data set.** Our analysis includes 8,855 solid tumor samples across 31 cancer types in the TCGA PanCancer Atlas collection that have the following data modalities: DNA alteration, RNA gene expression levels (RNA-seq), and clinical annotation. We exclude acute myeloid leukemia and adrenocortical carcinoma due to small sample sizes. To construct the undirected graph for the DNA alteration modality, we considered ten canonical oncogenic pathways: cell cycle, Hippo, Myc, Notch, Nrf2, PI-3-Kinase/Akt, RTK-RAS, TGF$\beta$ signaling, p53 and $\beta$-catenin/Wnt, which are frequently altered at the genetic (point mutations and copy number alterations) and epigenetic (epigenetic silencing) level, focusing on pathway members likely to be cancer drivers or therapeutic targets4. A total of 187 genes curated for these 10 signaling pathways are included for graph construction; and an edge is drawn between two genes if they belong in the same oncogenic pathway.

The 10 canonical signaling pathways with frequent genetic alterations include key cancer genes explored in pathways compiled by reviewing the full set of cancer type specific pathway diagrams from the compendium of TCGA manuscripts published between 2008 and 2017 [1]. The curation primarily focused on pathway members likely to be cancer drivers (functional contributors) or therapeutic targets. These pathway diagrams are publicly available as predefined network templates within the www.PathwayMapper.org visualization tool. The selected genes were then assessed for alterations within and across different tumor types. Alterations of pathway members were classified as activating events (typically recurrent missense mutations, i.e., hotspot mutations, amplifications, or fusions involving oncogenes) or inactivating events (truncating mutations, specific recurrent missense or inframe mutations, deletions, as well as fusions and promoter hypermethylation of tumor suppressor genes).

For mRNA gene expression data, we include 141 immune marker genes [2] and 103 genes with a stemness index [3], along with the top 1000 most variable genes (calculated using standard deviation). We used mRNA expression levels from the union of these genes (total of 1239). To construct networks among these, genes are connected if they belong to the same biological pathways (immune or stemness). Finally, We also consider between-modality biological interaction by connecting the same genes across modalities given the cis-regulation of gene expression. More complex between-modality edges could be incorporated based on additional biological knowledge depending on the study.

The 141 immune marker genes are the immune signature genes selected by Yoshihara et al. (2013) [2]. Briefly, gene expression profiles of normal hematopoietic samples were compared with those of other normal cell types in the GSE1133 data set. The overlap between the two gene sets constituted the immune signature. Genes associated with the quantity of infiltrating immune cells in tumor tissue were then identified using leukocyte methylation scores [4]. Genes in the high immune cell infil-

tration group were then overlapped with the normal hematopoietic cell-related genes. Furthermore, the Cancer Cell Encyclopedia (CCLE) expression data set was used to exclude genes with high variability across tumor types. This distillation process led to 141 immune marker genes which we used in our study. The process of selecting these 141 genes is described in detail in Yoshihara et al. (2013)[2].

The 103 stemness signature genes are derived in Malta et al. (2018) [3] by comparing pluripotent stem cells (ESC and iPSC) to stem cell-derived progenitors (EB, MESO, ECTO, DE) from PCBC and identified 87 CpGs that were hypomethylated in the pluripotent state (ESC and iPSC) compared to stem cell-derived progenitors and that potentially regulate the expression of 103 genes.

Clinical outcome data are taken from TCGA Pan-Cancer Clinical Data Resource (TCGA-CDR) [5], which originally contained 11,160 TCGA samples. The majority of samples are from primary solid tumors, while the 361 melanoma samples are from metastatic samples. For these metastatic samples, we use survival outcome calculated from the date of biospecimen collection/accession to the date of the last follow-up or death. DNA alteration data are downloaded from the Sanchez-Vega (2018) paper, which included 9125 TCGA samples from 33 cancer types. Upper quartile normalized RSEM data for batch-corrected mRNA gene expression were used for analysis; more details can be found in the TCGA PanCancer paper [5]. For mRNA gene expression data, we centered the data after log transformation. To accommodate missing values, we conduct multiple imputation with predictive mean matching method We directly impose the data into Nebula for analyses; and for iCluster, additional data processing is required for the DNA alteration input data: We filtered out genes with too few mutations for clustering, which left us with 41 genes whose mutation rate was $> 2\%$.

**Single cell RNA-seq data set.** The RNA-seq dataset is obtained from [6] profiling 68k peripheral blood mononuclear cells (PBMCs) from a healthy donor prepared on GemCode Single-Cell Instrument (10x Genomics, Pleasanton, CA, USA). Our analysis included normalized mRNA gene expression data from 10,310 genes in a total of 12,039 cells after filtering as in [7] and data pre-processing as described in [8].

# References

[1] Francisco Sanchez-Vega, Marco Mina, Joshua Armenia, Walid K Chatila, Augustin Luna, Konnor C La, Sofia Dimitriadoy, David L Liu, Havish S Kantheti, Sadegh Saghafinia, et al. Oncogenic signaling pathways in the cancer genome atlas. *Cell*, 173(2):321–337, 2018.

[2] Kosuke Yoshihara, Maria Shahmoradgoli, Emmanuel Martínez, Rahulsimham Vegesna, Hoon Kim, Wandaliz Torres-Garcia, Victor Treviño, Hui Shen, Peter W

Laird, Douglas A Levine, et al. Inferring tumour purity and stromal and immune cell admixture from expression data. *Nature communications*, 4(1):1–11, 2013.

[3] Tathiane M Malta, Artem Sokolov, Andrew J Gentles, Tomasz Burzykowski, Laila Poisson, John N Weinstein, Bożena Kamińska, Joerg Huelsken, Larsson Omberg, Olivier Gevaert, et al. Machine learning identifies stemness features associated with oncogenic dedifferentiation. *Cell*, 173(2):338–354, 2018.

[4] Scott L Carter, Kristian Cibulskis, Elena Helman, Aaron McKenna, Hui Shen, Travis Zack, Peter W Laird, Robert C Onofrio, Wendy Winckler, Barbara A Weir, et al. Absolute quantification of somatic dna alterations in human cancer. *Nature biotechnology*, 30(5):413–421, 2012.

[5] Jianfang Liu, Tara Lichtenberg, Katherine A Hoadley, Laila M Poisson, Alexander J Lazar, Andrew D Cherniack, Albert J Kovatich, Christopher C Benz, Douglas A Levine, Adrian V Lee, et al. An integrated tcga pan-cancer clinical data resource to drive high-quality survival outcome analytics. *Cell*, 173(2):400–416, 2018.

[6] Grace X Zheng, Jessica M Terry, Phillip Belgrader, Paul Ryvkin, Zachary W Bent, Ryan Wilson, Solongo B Ziraldo, Tobias D Wheeler, Geoff P McDermott, Junjie Zhu, et al. Massively parallel digital transcriptional profiling of single cells. *Nature Communications*, 8(14049), 2017.

[7] Michael B Cole, Davide Risso, Allon Wagner, David DeTomaso, John Ngai, Elizabeth Purdom, Sandrine Dudoit, and Nir Yosef. Performance assessment and selection of normalization procedures for single-cell rna-seq. *Cell Systems*, 8(4): 315–328, 2019.

[8] Romain Lopez, Jeffrey Regier, Michael B Cole, and Nir Yosef. Deep generative modeling for single-cell transcriptomics. *Nature Methods*, 15:1053–1058, 2018.
